# The behavioral and neural binding phenomena during visuomotor integration of angry facial expressions

**DOI:** 10.1038/s41598-018-25155-8

**Published:** 2018-05-02

**Authors:** Sélim Yahia Coll, Leonardo Ceravolo, Sascha Frühholz, Didier Grandjean

**Affiliations:** 1 Neuroscience of Emotion and Affective Dynamics’ laboratory, Department of Psychology and Educational Sciences and Swiss Centre for Affective Sciences, University of Geneva, Geneva, Switzerland; 2 Cognitive and Affective Neuroscience, Department of Psychology, University of Zurich, Zurich, Switzerland

## Abstract

Different parts of our brain code the perceptual features and actions related to an object, causing a binding problem, in which the brain has to integrate information related to an event without any interference regarding the features and actions involved in other concurrently processed events. Using a paradigm similar to Hommel, who revealed perception-action bindings, we showed that emotion could bind with motor actions when relevant, and in specific conditions, irrelevant for the task. By adapting our protocol to a functional Magnetic Resonance Imaging paradigm we investigated, in the present study, the neural bases of the emotion-action binding with task-relevant angry faces. Our results showed that emotion bound with motor responses. This integration revealed increased activity in distributed brain areas involved in: (i) memory, including the hippocampi; (ii) motor actions with the precentral gyri; (iii) and emotion processing with the insula. Interestingly, increased activations in the cingulate gyri and putamen, highlighted their potential key role in the emotion-action binding, due to their involvement in emotion processing, motor actions, and memory. The present study confirmed our previous results and point out for the first time the functional brain activity related to the emotion-action association.

## Introduction

The primate brain processes perceptual features (e.g., color, shape, and location) and motor actions related to an object in a distributed fashion, causing a binding problem^1^. In fact, how does the brain integrate the information belonging to one object without any interference from features pertaining to other concurrently processed objects? Hommel^2^ suggested the *event file* concept: A transient episodic memory trace binding features and motor actions pertaining to an event. In his paradigm, Hommel presented two geometric figures one after the other. These figures could be a circle or a cross, red or green, and located in the top or bottom part of the screen. Participants were instructed to respond to the first Fig. (S1) given the direction of a previous cue (right or left-pointing arrow). The response to the second Fig. (S2) depended on its shape, for the first experiment, and its color, for the second experiment. For instance, in the first experiment, they had to press the keyboard’s right arrow for a circle and the left for a cross, counterbalanced between 2 groups of participants. The assumption was that in S1, participants would bind in an event file their motor response with the features of the stimulus, and that in S2, their reaction time would vary depending on the repetition of the features and the motor response. They would be faster for a complete repetition, because the repeated element automatically triggers the others. They would also be faster for a complete alternation, because in this case no previous binding would disrupt the present response to S2. However, they would be slower when one element of S1 is repeated in S2, but not the others. In this partial repetition case, the binding has to be dissociated allowing a new response to S2, increasing the reaction time. Results showed that indeed participants were faster when the relevant feature and the motor response were both repeated and alternated than they were in the case of a partial repetition.

Following the results of Hommel^2^, three hypotheses were developed to explain the neural underpinnings of the event file^2,3^: The integration by convergence, indexing, and correlation. The integration by convergence assumption is based on the grandmother cell hypothesis^4^, i.e. the idea that our brain contains neurons specialized in the binding of specific features. For example, we would have cells responding to red squares, green triangles or even more complex structures like the face of our grandmothers. This hypothesis is interesting, and cells responding to the conjunction of different features were in fact found in the visual cortex^5^, but it is unlikely that the integration by convergence would be the only mechanism of the event file. Indeed, we would need as many cells as there is of combinations in the environment, causing an obvious space problem. The integration by indexing and correlation assumptions resolve this dilemma by having different brain areas coding the perceptual and motor information. In the former case, the link is made by enhancing the firing rates of the neural units, and in the latter case, by synchronizing the firing patterns^1^. Thus, although the integration by indexing creates a binding based on the quantity of the exchanges, the integration by correlation build it based on the quality. Interestingly, many recent evidences seem to favor the latter process, also called binding-by-synchronization (BBS) hypothesis^3,6^, because it has the advantage of being flexible and dynamic, as well as facilitating the neural transmission over widely separated areas of the brain^3^. To our knowledge, only one study adapted Hommel’s paradigm^2^ to a functional Magnetic Resonance Imaging (fMRI) protocol to investigate the neural processes implicated in the event file. Kuhn and colleagues^7^, using photographs of faces (neutral expression) and houses, focused their analyses on four brain regions: The fusiform face area (FFA), the parahippocampal gyrus (PPA), and the right and left motor cortices. They then discovered that in a partial repetition case, the brain area processing the feature of the event file that was alternated from S1 to S2, showed a reduced activation at the time of S2 in order to allow a new binding.

Following these findings, several authors investigated the application of the event file using emotional contents^8–12^. Indeed, the event file could be the main mechanism underlying phobia, obsessive-compulsive disorders or the post-traumatic stress syndrome: A stimulus previously associated with a traumatic event reactivating an intense fear or panic moment. Moreover, in the general population, an automatic and accurate response to emotional events was probably favored during our ontogenetic and phylogenetic history in order to adapt to our environment. Therefore, authors developing theories of emotion^13,14^ emphasized the link between emotions and actions through the concepts of action tendencies^15,16^, emotion embodiment^17^, or motivational theory^18^. Lavender and Hommel^19^ (2007), following the path of several researchers^20^, used an approach-avoidance protocol to highlight a valence-action binding, showing that participants moving a doll toward positive pictures and away from negative pictures were faster than participants with opposite instructions, a result probably due to overtrained approach-positive and avoidance-negative associations.

In a previous series of studies^21^, we replaced the geometric figures of Hommel^2^ by faces expressing anger, fear or no emotions (neutral expression) to investigate a possible emotion-action association. Our choice of adapting Hommel’s paradigm was based on the fact that it has several relevant characteristics: (i) It introduces a motor response (R1) to S1 allowing the investigation of perceptive-motor bindings (i.e. event file bindings). (ii) S1 and R1 occur in close temporal proximity favoring their integration into a single event file. (iii) S1 is the only stimulus displayed, preventing from integrating a distractor or alternative stimulus. (iv) R1 is signaled by a stimulus other than the prime (a cue taking the form of a right or left-pointing arrow), allowing orthogonal variation of the features and motor response in S1. When the task in S2 was an emotional task (decision between emotion and neutral), participants were faster for a complete repetition and alternation of the emotion and motor response than for a partial repetition of them, expanding results of Hommel to the integration of emotional faces. Moreover, our results seemed to highlight a different influence of emotional stimuli and neutral stimuli in their binding with motor responses. Participants were faster for an emotion and motor response repetition when emotional faces rather than neutral faces were repeated. They were also faster for a motor response repetition, but an emotion alternation if S1 was neutral rather than angry. When S1 is neutral, participants have to dissociate the motor response from a neutral face at the time of S2 and associated it to an emotional expression. From our results, it would seem that this situation is less costly in reaction time. It may be due to the fact that a binding implicating emotional expressions in S1 is more difficult to dissociate at the time of S2 than a binding implicating neutral features^21^.

In line with our results, Fitousi^22,23^, using the same adaptation of Hommel’s paradigm^2^, but presenting photographs of people expressing sadness and anger, investigated the binding of several facial features (race, identity, age, etc.), including emotion. The author’s prediction was that like simple dimensions (e.g., shape and color), facial attributes are processed in a distributed fashion in the brain^24^. For instance, emotion is processed in the amygdala and other brain areas (e.g., insula) and structures (e.g., basal ganglia) that have been largely documented in emotion research, whereas personal identity is processed in the anterior temporal region^24^. Furthermore, recordings in the temporal cortex of non-human primates have revealed a distributed neuronal activity when processing faces^25^. Thus, Fitousi^22,23^ revealed that not only emotion, but facial features in general could take part in bindings, that he suggested calling *face files* when dealing with faces.

The existence of an emotion-action binding was highlighted in the literature, but these experiments do not allow us to conclude on the neural bases of such integration. One way to do this would be to use our adaptation of Hommel’s paradigm^21^ and to modify the timings to the requirements of the fMRI^7^. One could then compare the activation of brain areas implicated in a complete repetition and alternation of the emotion and motor responses to the activation of a partial repetition of them. Several brain areas would be expected to play a role in the emotion-action integration. Firstly, according to the event file theory, areas implicated in the memory encoding and retrieval, such as the hippocampus and/or surrounding areas^1^. Secondly, areas involved in the emotion processing, that is to say limbic areas, such as the insula or amygdala^26^. Thirdly, motor areas associated to the right and left index finger responses. Finally, two regions might play a key role in the emotion-action binding: The cingulate gyri and the basal ganglia. The cingulate gyrus has been shown to play several functions closely related to the integration of emotional stimuli with motor responses. Although the cingulate gyrus is classically known for its implication in emotion processing^27,28^, studies also emphasized its implication in motor actions, memory, conflict monitoring and inhibition^27–32^. The basal ganglia are known for their involvement in pattern movement generation, in particular with all the research with Parkinson’s disease patients^33^. However, more recent reviews also highlighted their role in the procedural memory and habits^34^, as well as emotion processing^35^. In fact, several researchers suggested a segregation of the basal ganglia in three functional roles: An emotional, motor, and associative role^36,37^. These aspects would be supported by different neuronal groups with many interconnections, suggesting an integrative function of the basal ganglia^38,39^. Basal ganglia could then be a key area in the visuomotor binding implicating emotional events.

Our present experiment was then developed to go further on our previous behavioral findings by investigating the neural underpinnings of the emotion-action binding using an fMRI. Our behavioral assumption stemmed directly from the previous study: We expected an emotion-action binding; we then assumed that participants would be faster for a complete repetition and alternation of the emotion and motor response than for a partial repetition of them. Moreover, we predicted a different influence of emotional stimuli and neutral stimuli in this binding: When the emotion and motor response were both repeated from S1 to S2, we expected participants to be faster for a repetition of angry faces than neutral faces. Furthermore, in the case of a motor response repetition, but emotion alternation, we assumed participants to be faster when the first face was neutral rather than angry.

Concerning neuroimaging, we predicted a greater activity for a complete repetition and alternation of the emotion and motor response than for a partial repetition of them in the brain areas implicated in the memory encoding and retrieval, emotion processing, and motor actions (see above).

## Results

### Behavioral results

**Figure 1:**
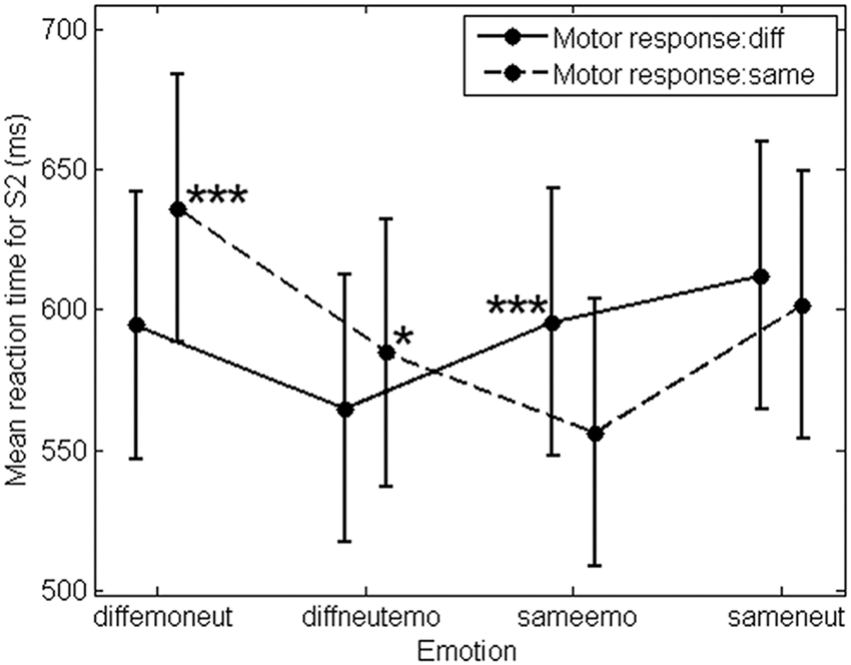
Behavioral analyses. Interaction between emotion and motor response. Stars represent simple effects’ results comparing a motor response repetition to a motor response alternation for each level of the emotion factor. ***Corresponds to a significance level of 0.001 and *To a significance level of 0.05. Vertical bars represent confidence intervals of 0.95.

For information purposes, main effects and accuracy results can be found in the Appendix A and B of the supplementary materials section. Concerning our assumptions, we hypothesized that emotion would integrate an event file, indeed as shown in Fig. 1 emotion significantly interacted with the motor response, *F*(3, 2152) = 10.23, *p* < 0.001, *R*^2^_*m*_ = 0.03, *R*^2^_*c*_ = 0.32 (Note: *R*^2^_*m*_ represents the variance explained by the fixed factors, while *R*^2^_*c*_ is the variance explained by both fixed and random effects^40^). Simple effects showed that participants were significantly faster for a motor response alternation than repetition in the diffemoneut, χ^2^(1, *N* = 19) = 14.98, *p* < 0.001, and diffneutemo, χ^2^(1, *N* = 19) = 4.73, *p* = 0.03, conditions. Moreover, they were significantly faster for a motor response repetition than variation in the sameemo, χ^2^(1, *N* = 19) = 11.02, *p* < 0.001, but not sameneut condition, χ^2^(1, *N* = 19) = 0.84, *p* = 0.36.

We also hypothesized a different influence of emotional and neutral events in the emotion-action binding. Our results showed that participants were faster for an emotion repetition when the motor response was repeated, if both faces were angry rather than neutral, χ^2^(1, *N* = 19) = 23.12, *p* < 0.001. Moreover, when the motor response was repeated, but not the emotion, participants were faster when the first face was neutral compared to the situation with an angry face in S1, χ^2^(1, *N* = 19) = 18.83, *p* < 0.001.

### Image results

#### Finger localizer task

A summary of the finger localizer image results can be found in Table 1. In order to locate the motor areas associated to the index finger responses and use them as a region of interests (ROI) in the binding task analyses (see Appendix C in the supplementary materials section), a finger localizer task was performed by the participants. Results showed that a right motor response activated more than a left motor response the right cerebellum anterior lobe, the left parietal postcentral gyrus, the right cerebellum posterior lobe and the left thalamus. On the opposite, a left motor response activated more than a right motor response the right frontal precentral gyrus, the left cerebellum anterior lobe, the right insula, the right putamen and the left occipital superior lobe.

**Table 1:** Summary of the finger localizer image results. Finger localizer contrasts were thresholded at *p* < 0.001 (uncorrected) with a cluster extent of *k* = 36, corresponding to a Family-Wise Error (FWE) correction for multiple comparisons of *p* < 0.05 at the cluster level.

Right > left					
area	x	y	z	*t*	cluster size
right cerebellum anterior lobe	12	−48	−16	8.51	616
left parietal postcentral gyrus	−40	−20	54	7.88	712
right cerebellum posterior lobe	8	−62	−36	5.03	165
left thalamus	−18	−24	6	4.83	50
**Left > right**					
**area**	**x**	**y**	**z**	***t***	**cluster size**
right frontal precentral gyrus	36	−18	48	10.23	1562
left cerebellum anterior lobe	−16	−46	−20	7.98	809
right insula	38	−14	22	5.09	186
right putamen	32	−4	0	5	48
left occipital superior lobe	−10	−100	8	4.8	40

#### Binding task: General emotion effect

A summary of the binding task image results can be found in Table 2. Emotional faces in S2 activated more than neutral faces the right parietal postcentral gyrus, the right frontal precentral gyrus and the right insula. Including the left > right finger localizer contrast as ROI showed an activation of areas coding the right index finger motor responses.

**Table 2:** Summary of the binding task image results. Binding task contrasts were thresholded at *p* < 0.001 (uncorrected) with a cluster extent of *k* = 23, corresponding to a Family-Wise Error (FWE) correction for multiple comparisons of *p* < 0.05 at the cluster level.

Emotion > neutral					
area	x	y	z	*t*	cluster size
right parietal postcentral gyrus	56	−14	16	4.63	73
right frontal precentral gyrus	28	−16	68	4.62	30
right frontal precentral gyrus	58	−14	44	4.5	73
right frontal precentral gyrus	48	−10	52	4.33	46
right parietal postcentral gyrus	26	−34	68	4.09	69
right insula	38	−20	16	4.02	28
**Complete repetition + alternation > partial repetition**					
**area**	**x**	**y**	**z**	***t***	**cluster size**
right frontal sub-gyral	18	−14	58	4.1	24
right frontal precentral gyrus	56	−12	34	4.09	44
left middle temporal gyrus	−48	−62	2	4.08	60
right cingulate gyrus	14	−42	30	4.21	55
**Complete repetition > partial repetition**					
**area**	**x**	**y**	**z**	***t***	**cluster size**
right medial frontal gyrus	6	−28	72	3.75	25
left parahippocampal gyrus	−24	−28	−14	4.32	61
right hippocampus	22	−30	−10	3.67	23
left putamen	−16	12	−10	4.32	97
right middle temporal gyrus	50	0	−24	4.31	39
left anterior cingulate gyrus	−18	34	18	4.08	24
right parietal precuneus	6	−80	40	4.27	72
**Complete alternation > partial repetition**					
**area**	**x**	**y**	**z**	***t***	**cluster size**
left middle temporal gyrus	−54	−64	2	3.67	26
**Emotional > neutral stimuli influence in the binding**					
**area**	**x**	**y**	**z**	***t***	**cluster size**
right frontal precentral gyrus	28	−32	62	3.82	72
right frontal precentral gyrus	48	−12	10	4.08	168
left precuneus	−14	−54	64	3.73	26
right transverse gyrus	52	−26	12	3.99	31
left insula	−38	−12	4	4.44	32

#### Binding task: Emotion-action binding effect

**Figure 2:**
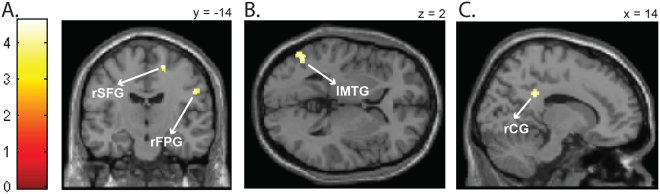
Emotion-action binding effect. Brain areas significantly (*p* < 0.001, cluster corrected at *p* < 0.05) more activated for a complete repetition and alternation of the emotion and motor response than for a partial repetition of them, as shown by the *complete repetition* + *alternation* > *partial repetition* t-contrast. (**A**) Activation of the right superior frontal gyrus (rSFG) and right frontal precentral gyrus (rFPG). (**B**) Activation of the left middle temporal gyrus (lMTG). (**C**) Activation of the right cingulate gyrus (rCG).

A complete repetition and alternation of the emotion and motor response activated more than a partial repetition the right superior frontal gyrus (Fig. 2A), the right frontal precentral gyrus (Fig. 2A), the left middle temporal gyrus (Fig. 2B) and the right cingulate gyrus (Fig. 2C). Interestingly, including the left> right finger localizer contrast as ROI interest showed an activation of areas coding the right index finger motor responses.

**Figure 3:**
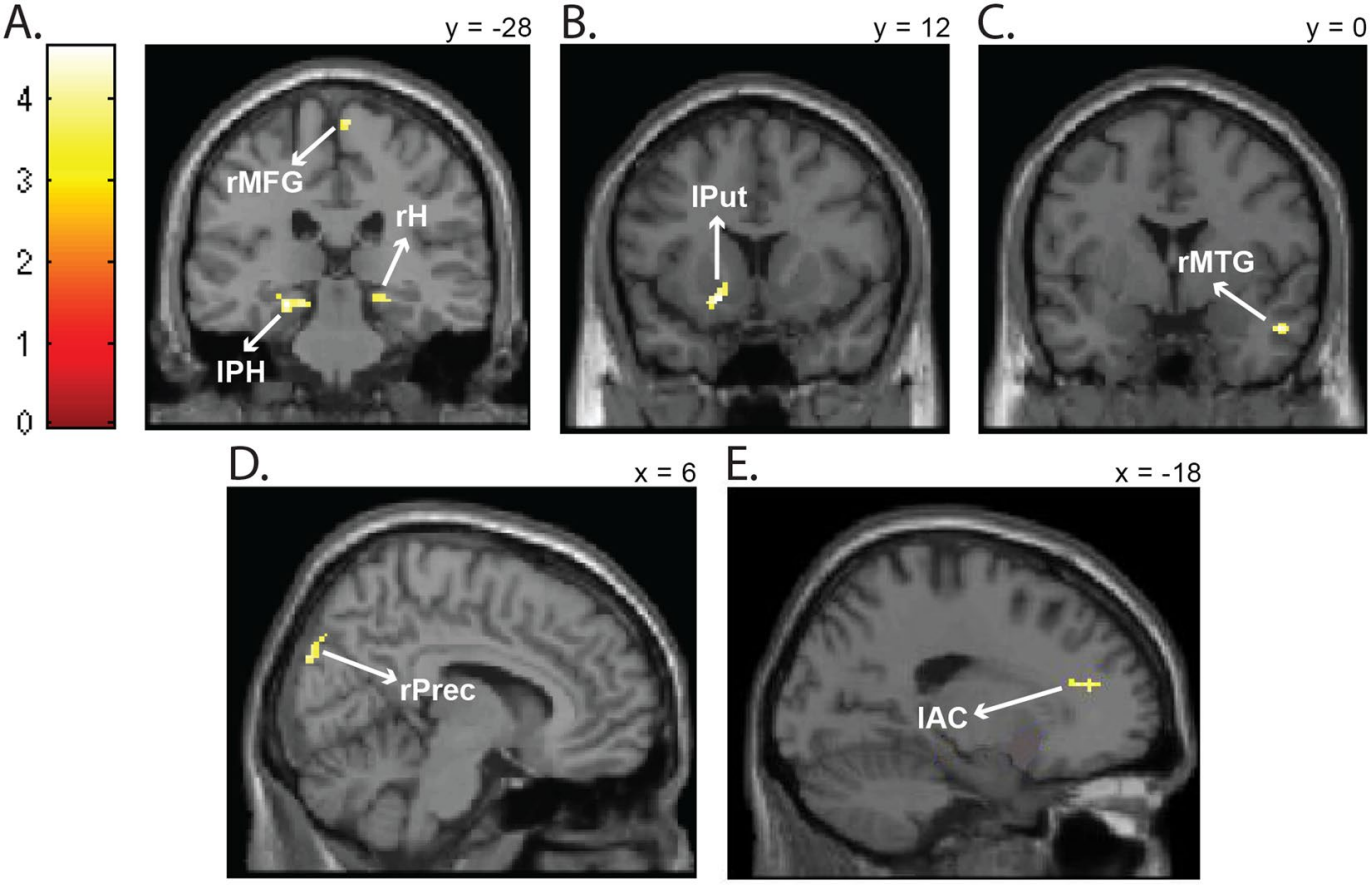
Emotion-action binding effect. Brain areas significantly (*p* < 0.001, cluster corrected at *p* < 0.05) more activated for a complete repetition of the emotion and motor response than for a partial repetition of them, as shown by the *complete repetition* >*partial repetition* t-contrast. (**A**) Activation of the right medial frontal gyrus (rMFG), the left parahippocampal gyrus (lPH) and the right hippocampus (rH). (**B**) Activation of the left putamen (lPut). (**C**) Activation of the right middle temporal gyrus (rMTG). (**D**) Activation of the right precuneus (rPrec). (**E**) Activation of the left anterior cingulate gyrus (lAC).

A complete repetition activated more than a partial repetition the right medial frontal gyrus (Fig. 3A), the left parahippocampal gyrus (Fig. 3A), the right hippocampus (Fig. 3A), the left putamen (Fig. 3B), the right middle temporal gyrus (Fig. 3C), the right precuneus (Fig. 3D) and the left anterior cingulate gyrus (Fig. 3E).

Finally, a complete alternation activated more than a partial repetition the left middle temporal gyrus.

#### Binding task: Emotional vs. neutral stimuli influence in the binding

**Figure 4:**
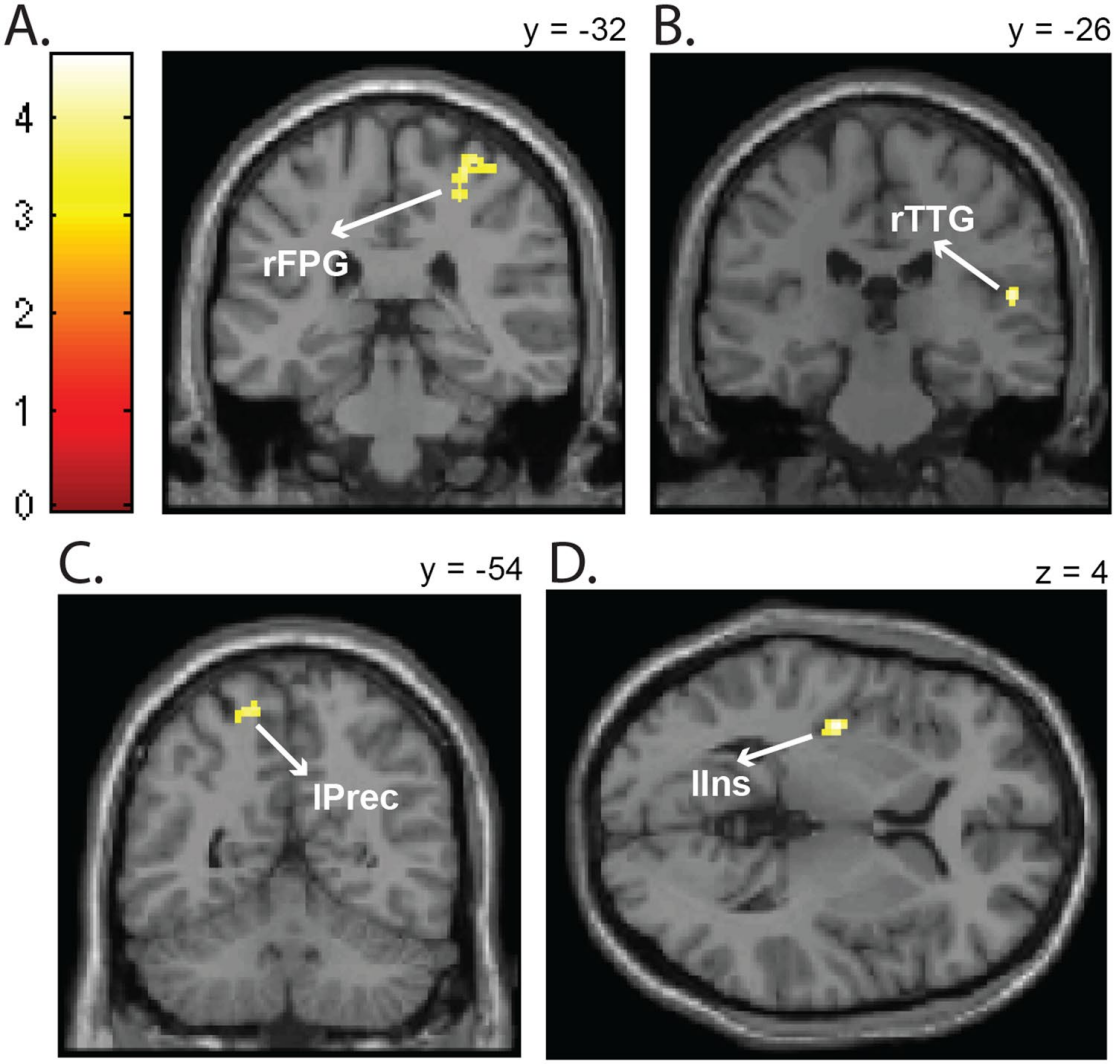
Emotional vs. neutral stimuli influence in the binding. Brain areas significantly (*p* < 0.001, cluster corrected at *p* < 0.05) more activated for a complete repetition of emotional than neutral faces when the motor response is also repeated, as shown by the *same response* + *sameemotion* > *same response* + *sameneut* t-contrast. (**A**) Activation of the right frontal precentral gyrus (rFPG). (**B**) Activation of the right transverse temporal gyrus (rTTG). (**C**) Activation of the left precuneus (lPrec). (**D**) Activation of the left insula (lIns).

In the case of a complete repetition of the emotion and motor response, a repetition of emotional faces activated more than a repetition of neutral faces the right frontal precentral gyrus (Fig. 4A), the right transverse temporal gyrus (Fig. 4B), the left precuneus (Fig. 4C) and the left insula (Fig. 4D). Including the left> right finger localizer contrast as ROI showed an activation of areas coding the right index finger motor responses.

#### Binding task: Functional connectivity

Concerning our t-contrasts of interest, the *emotion* > *neutral*, the *complete repetition* > *partial repetition*, and the *complete alternation* > *partial repetition* contrasts did not show any significant correlations between our ROIs. However, the *complete repetition* + *alternation* > *partial repetition* contrast revealed a significant negative correlation between the left putamen as a seed region and the right hippocampus, *t*(18) = −3.32, *p* < 0.05 (FDR-corrected). Moreover, the *same response* + *sameemotion* >*same response* + *sameneut* contrast highlighted a significant negative correlation between the left middle temporal gyrus as a seed region and the left parietal postcentral gyrus, *t*(18) = −3.73, *p* < 0.05 (FDR-corrected).

**Figure 5:**
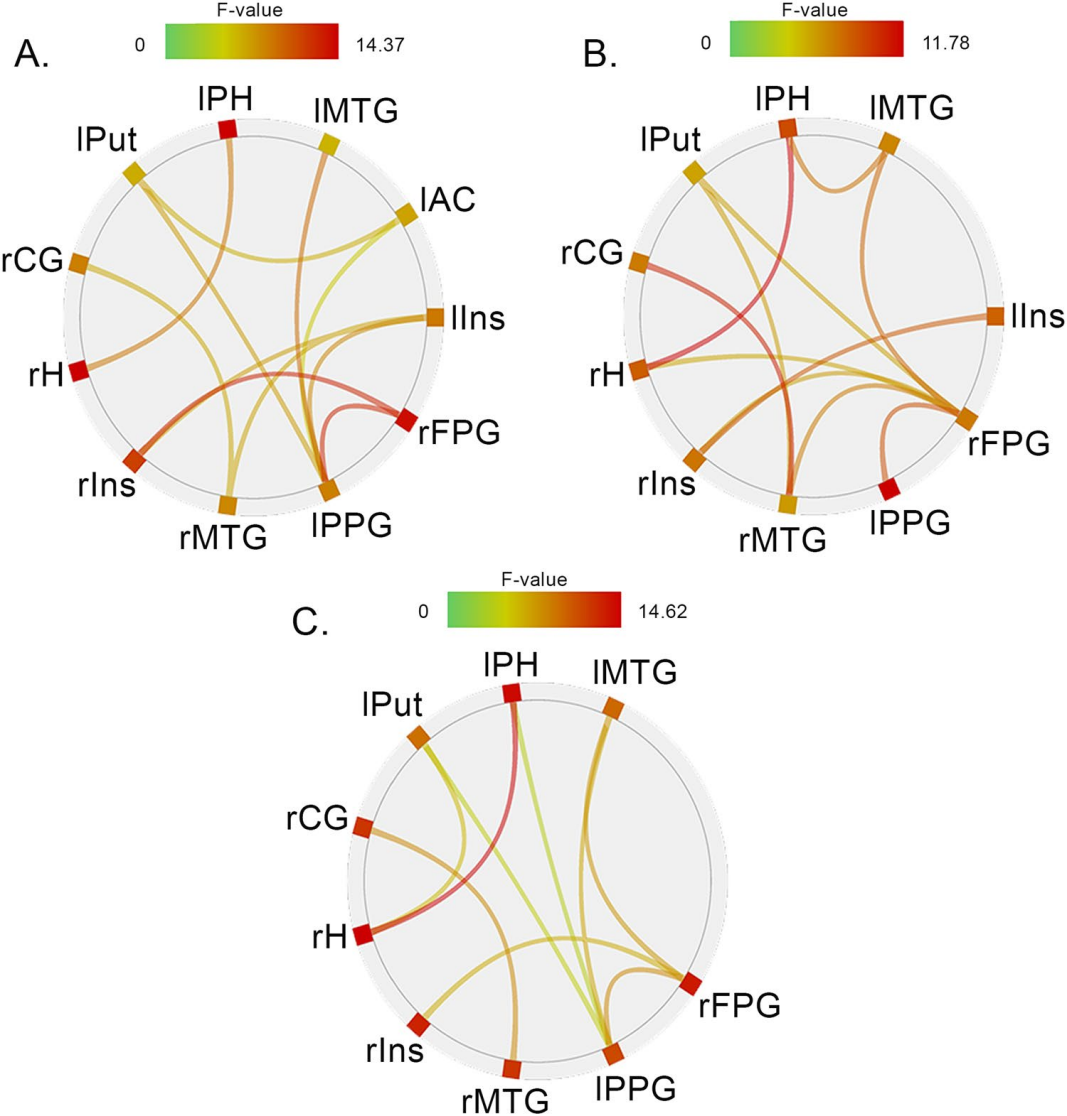
Functional connectivity analysis. FDR corrected (<0.05 at the seed level) correlations for (**A**) A complete repetition, (**B**) A complete alternation and (**C**) A partial repetition of the emotion and motor response in the binding task between our 11 ROIs of interest: The left parahippocampal gyrus (lPH), the left middle temporal gyrus (lMTG), the left anterior cingulate gyrus (lAC), the left insula (lIns), the right frontal precentral gyrus (lFPG), the left parietal postcentral gyrus (lPPG), the right middle temporal gyrus (rMTG), the right insula (rIns), the right hippocampus (rH), the right cingulate gyrus (rCG) and the left putamen (lPut).

Concerning the F-contrasts (see Fig. 5), a complete repetition of the emotion and motor response revealed several positive correlations between our ROIs: (i) The activity in the right frontal precentral gyrus correlated with the activity in the left parietal postcentral gyrus and the right insula, (ii) the left putamen with the left parietal postcentral gyrus and the left anterior cingulate gyrus, (iii) the left insula with the left parietal postcentral gyrus and the right insula, (iv) the left middle temporal gyrus with the left parietal postcentral gyrus, (v) the left parietal postcentral gyrus with the right frontal precentral gyrus, the left middle temporal gyrus, the left putamen, the left insula, and the left anterior cingulate gyrus, (vi) the right insula with the right frontal precentral gyrus and the left insula, (vii) the right middle temporal gyrus with the left insula and the right cingulate gyrus, and (viii) the left parahippocampal gyrus with the right hippocampus.

A complete alternation of the emotion and motor response also showed significant positive correlations: (i) The activity in the left parietal postcentral gyrus correlated with the activity in the right frontal precentral gyrus, (ii) the right middle temporal gyrus with the right cingulate gyrus, the right frontal precentral gyrus, and the left putamen, (iii) the right frontal precentral gyrus with the left middle temporal gyrus, the left parietal postcentral gyrus, the right insula, the right middle temporal gyrus, the left putamen, and the right hippocampus, (iv) the right hippocampus with the left parahippocampal gyrus, (v) the left parahippocampal gyrus with the right hippocampus, (vi) the right cingulate gyrus with the right middle temporal gyrus, (vii) the left middle temporal gyrus with the right frontal precentral gyrus and the left parahippocampal gyrus, and (viii) the right insula with the left insula.

Finally, a partial repetition highlighted significant positive correlations as well: (i) The activity in the left parahippocampal gyrus correlated with the activity in the right hippocampus, (ii) the right hippocampus with the left parahippocampal gyrus and the left putamen, (iii) the right insula with the right frontal precentral gyrus, (iv) the right cingulate gyrus with the right middle temporal gyrus, (v) the left parietal postcentral gyrus with the right frontal precentral gyrus, the left middle temporal gyrus, the left putamen, and the left parahippocampal gyrus, (vi) the right frontal precentral gyrus with the left parietal postcentral gyrus, the left middle temporal gyrus, and the right insula, (vii) the left putamen with the right hippocampus, (viii) the right middle temporal gyrus with the right cingulate gyrus, and (ix) the left middle temporal gyrus with the right frontal precentral gyrus and the left parietal postcentral gyrus.

## Discussion

The aim of the present study was to investigate the implication of emotion-action binding at the neural level. Using a timing-adapted version of our previously published paradigm^21^, we obtained at the behavioral level a significant interaction between the emotion and motor response, in which emotional and neutral faces influenced the emotion-action binding differently. At the neural level, a complete repetition and/or alternation of the emotion and motor response compared to a partial repetition of these elements enhanced activity in the right hippocampus, the left parahippocampal gyrus, the middle temporal gyrus bilaterally, the insula, the right frontal precentral gyrus, the bilateral cingulate gyrus, the precuneus, and the left putamen.

The present study successfully highlighted an emotion-action binding. Indeed, as expected based on our previous series of studies^21^ and the study of Fitousi^23^, participants were faster for a complete alternation than partial repetition of the emotion and motor response. Moreover, they were faster for a repetition of emotional faces than for a partial repetition, when the motor response was also repeated. However, contrary to our prediction, a repetition of neutral faces did not yield similar results. One possible explanation is the fact that the timing of our present study had to be modified to fit the requirements of fMRI. Indeed, in order to leave enough time for the hemodynamic response to recover, we increased the timing compared to our previous work, and in particular the fixation cross between S1 and S2^21^. Thus, although participants had a 500-ms interval between the faces in the previous experiments, they had an interval of 1000 to 3000 ms in the present study. A greater interval might have reduced our effects by leaving more time for the association created in S1 to fade. Interestingly, however, the fact that in a complete repetition case the effect disappeared when neutral but not emotional faces were repeated, could emphasize a stronger impact of emotional faces in the emotion-action binding. This assumption is in accordance with studies comparing neutral and emotional events regarding perception, attention or memory^41–43^, showing an advantage for emotional information due to their relevance for our biological survival. In the same vein, our simple effects revealed that participants were faster for a complete repetition of the emotion and motor response, when emotional faces rather than neutral faces were repeated. When the motor response was repeated, but not emotion, participants were slower when the face in S1 was angry rather than neutral. These results can be interpreted as if the binding in S1 was greater for emotional faces than neutral faces, facilitating the response in S2 in a complete repetition situation, but being costlier to break in a partial repetition case. However, we could also explain the different influence of emotional and neutral stimuli at the level of S2 only. From this perspective, our results would be interpreted as differences in retrieval between emotional and neutral faces or just as differences between their processing. We also cannot exclude that all these effects operate conjointly. The fact that binding effects are always tested in sequential paradigms makes it indeed difficult to distinguish between these possible competing processes.

Concerning our fMRI analyses, angry faces compared to neutral expressions triggered enhanced activity in the right parietal postcentral gyrus and the right frontal precentral gyrus. These two areas, as shown by including the results of the contrasts of the finger localizers as ROIs, are also involved in the motor response. Interestingly, these results highlight once again the way emotional stimuli are strongly associated with motor responses. Related to emotional processing, the right insula was also more activated for angry compared to neutral faces, a fact that is more commonly observed in the literature^44–46^.

Concerning effects of the emotion-action binding, we demonstrated, as predicted, that a complete repetition and alternation of the emotion and motor response, compared to partial repetitions, enhanced activity in brain regions known for their involvement in memory encoding and retrieval, including the left parahippocampal gyrus, the right hippocampus^47^, as well as the right parietal precuneus^48^. The middle temporal gyri, whose involvement has been highlighted in semantic knowledge^49–51^ and face processing^52–55^, were also more activated. Significant increases were observed in the right frontal precentral gyrus and the left putamen, implicated in motor patterning and actions^33^. Interestingly, the left putamen, as part of the basal ganglia, could be an important structure in emotion-action binding. Indeed, its activation is not only associated with motor responses, but also with emotion processing^35^, habits and procedural memory^34^. Moreover, the integrative role of the basal ganglia has also been acknowledged by researchers, particularly in the model of Haber^38^, in which different loops associated with one of the motor, limbic, and associative function of the basal ganglia, communicate with each other in order to modulate behavior. Another interesting area, which was activated in the current binding task, is the cingulate gyrus (complete repetition and alternation of the emotion and motor response > partial repetition). The cingulate gyrus might play an important role in emotion-action binding due to its implication in emotion processing, motor actions, memory, conflict monitoring and inhibition^27–32^. When comparing the repetition of emotional vs. neutral faces in the situation of a complete repetition of the emotion and motor response, we demonstrated a greater activation of the right frontal precentral gyrus, implicated in the motor response, but also an activation of the left insula. This latter is particularly interesting, because it echoes the result obtained above when studying the general activation of emotional expressions (*emotion* > *neutral* contrast). Indeed, the right frontal precentral gyrus and the right part of the insula were more activated in general for emotional faces compared to neutral faces. The greater impact of emotional faces compared to neutral faces in emotion-action binding, observed at the behavioral level could then be the result of the stronger activation of these areas for emotional events.

Finally, in order to better target the link between the different brain areas activated during the binding task, which is one of the most important hypothesis in the context of binding, i.e. different brain regions have to be in transient interactions to build up percepts or to achieve actions on percepts (see the BBS hypothesis^3,6^), we decided to perform a functional connectivity analysis. The analysis comparing the activity for a complete repetition and alternation of the emotion and motor response to a partial repetition of them highlighted a significant negative correlation between the left putamen as a seed region and the right hippocampus. Moreover, comparing the effects of an emotional binding with the motor response to those of a neutral binding showed a negative correlation between the left middle temporal gyrus as a seed region and the left parietal postcentral gyrus. These results emphasize the importance of two areas involved in motor action: The putamen^33^ and the left parietal postcentral gyrus. As stated above, putamen implication was also observed in emotion processing^35^, habits and procedural memory^34^, making it a possible key area for the emotion-action binding. The more these regions were activated in S2, the less were the right hippocampus, involved in memory encoding and retrieveal^43^, and the left middle temporal gyrus, implicated in semantic knowledge^49–51^ and face processing^52–55^, when the binding was favored (i.e. in a complete repetition and alternation case, rather than in a partial repetition situation, and when emotional stimuli instead of neutral stimuli were repeated).

In accordance with the assumption of synchronized firing patterns coming from different specialized brain areas (i.e. BBS hypothesis^3,6^), the F-contrasts revealed several positive connectivity in the binding task. On one side, the hippocampal areas (i.e., the right hippocampus and the left parahippocampal gyrus, both implicated in memory processing^47^) were strongly correlated, and on the other, the areas activated in motor actions (i.e. the right frontal precentral gyrus, the left parietal postcentral gyrus, and the left putamen). Interestingly, brain regions from both sides also correlated with each other. In fact, areas involved in motor patterning and actions, i.e. an important dimension of the event file, showed positive correlations with the middle temporal gyri implicated in semantic knowledge^49–51^ and face processing^52–55^, and the right cingulate gyrus. The latter region, as for the putamen, with which a positive correlation was obtained, might be a crucial area for emotion-action binding due to its activation in emotion processing, motor actions, and memory^27,28,30,32^. Finally, in agreement with our observations comparing the impact of emotional faces and neutral faces in binding, our functional connectivity results revealed strong interactions between the right frontal precentral gyrus and the right insula (itself functionally connected to the left insula). Interestingly, this link seemed even stronger in a complete repetition case, when all the contingencies were repeated from S1 to S2, which could potentially suggest a central role of the relation between these two regions in emotion-action integration.

To the best of our knowledge, only a single study investigated the functional activity related to the event file using the paradigm of Hommel^2^. Authors restricted their analyses to the difference in activation from S1 to S2 of four brain regions (FFA, PPA, and bilateral motor cortices). With the present experiment we wanted to shed new light on this general binding phenomenon by comparing the activity due to a complete repetition and alternation of the contingencies (i.e. the situations in which the binding is favored) to a partial repetition of them (i.e. the situation in which the binding is dissociated) without restricting our analyses to a priori ROIs and studying the functional connectivity. Moreover, we were interested in investigating specifically the binding of emotional stimuli with motor responses, a topic little dealt with in the literature, although having a great impact on our functioning (normal and pathologic, for example, as stated in the introduction, in anxiety disorders). We were able not only to confirm previous behavioral findings on the integration of facial features^22,23^, but we further emphasized the impact of emotion on the perception-action binding, only shown behaviorally in our previous work^21^, and highlighted functionally at the brain level in the present experiment. Thanks to functional connectivity analyses we could also demonstrate that binding effects implicate different brain areas that correlate their activity more or less strongly depending on whether the binding is in a favorable situation (i.e. complete repetition and alternation) or not (i.e. partial repetition), in accordance with the BBS integration assumption^3,6^.

## Conclusions and Future Directions

At the behavioral level, an emotion-action binding was observed, and this integration seemed to be differently influenced by emotional and neutral stimuli. At the neural level, we highlighted the role of areas involved in the memory encoding and retrieval (the right hippocampus and left parahippocampal gyrus), emotion processing (the insula), face processing and semantic knowledge (the middle temporal gyri), as well as motor actions (the right precentral gyrus). Moreover, we discovered enhanced activity in the basal ganglia and the cingulate gyri, whose implication in emotion processing, motor actions, and memory could suggest a key role in the emotion-action binding. With our present study, we were able to confirm the existence of the emotion-action binding and to study its mechanism at the neural level. The next step could be to investigate more precisely the functional organization of the brain areas highlighted by our binding task and in particular the role of the basal ganglia and the cingulate gyri, by recording for example intracranial data in this area in implanted Parkinson’s disease and pharmaco-resistant epileptic patients benefiting from a surgery. One could also further investigate the timing aspects of the emotion-action binding, which particularly influenced the integration of the neutral faces in the present experiment. It could be done by varying more systematically the interval between S1 and S2 or other elements of the paradigm, such as the presentation time of S1. Using a high temporal resolution technique like electroencephalography could also be a solution to disentangle these aspects.

## Methods

### Participants

Twenty participants (13 men; *M*_*age*_ = 26 years, *SD* = 6.69) from the University of Geneva and surroundings took part in this study. The sample size was decided based on a power analysis realized on a previous behavioral experiment^21^ with the “simr” package on R^56^. Indeed, 100% of power (95% CI, [99.63, 100]) for an effect size of *R*^2^_*m*_ = 0.03 and *R*^2^_*c*_ = 0.34 was obtained with 20 participants. We also note that Kuhn and colleagues^7^ used 21 participants for their fMRI study of the event file. Moreover, as shown by Desmond and Glover^57^, about twenty participants is also sufficient for a neuroimaging study, as estimated for a conservative alpha [0.01 < α < 0.05] and accounting for at least 80% of the variance at the single voxel level. All participants reported normal or corrected-to-normal vision and were not familiar with the purpose of the experiment. Written informed consent was obtained from all participants. The experiment was conducted at the Brain and Behavior Laboratory of the University of Geneva according to the declaration of Helsinki and approved by the Geneva research cantonal ethical committee (CCER; http://ge.ch/sante/commission-cantonale-dethique-de-recherche-ccer/commission-cantonale-dethique-de-recherche-ccer). One participant (1 male participant) was excluded from the analyses due to excessive movement artifacts.

### Materials and trial sequence

The experiment consisted of 2 tasks described in detail below.

#### Binding task

The stimulus material consisted of 3D grayscaled avatar faces of 2 men and 2 women expressing anger or being neutral. They were elaborated with the FACSGen software^58,59^ and were selected on the basis of the study of Roesch and colleagues^59^, in which 44 participants evaluated them with 69 other FACSgen stimuli in terms of gender, believability, and intrinsic emotionality. In the same study they were also rated by 20 participants in terms of emotion intensity on a scale from 0 (”not intense”) to 100 (”very intense”). Results of Roesch and colleagues^59^ showed that the mean intensity ratings for their angry and neutral faces were 60 (*SD* = 5.52) and 20.8 (*SD* = 5.52), respectively. In order to minimize low-level confounds, the contrast and luminance histograms of the pictures were equalized using the Matlab SHINE toolbox^60^.

**Figure 6:**
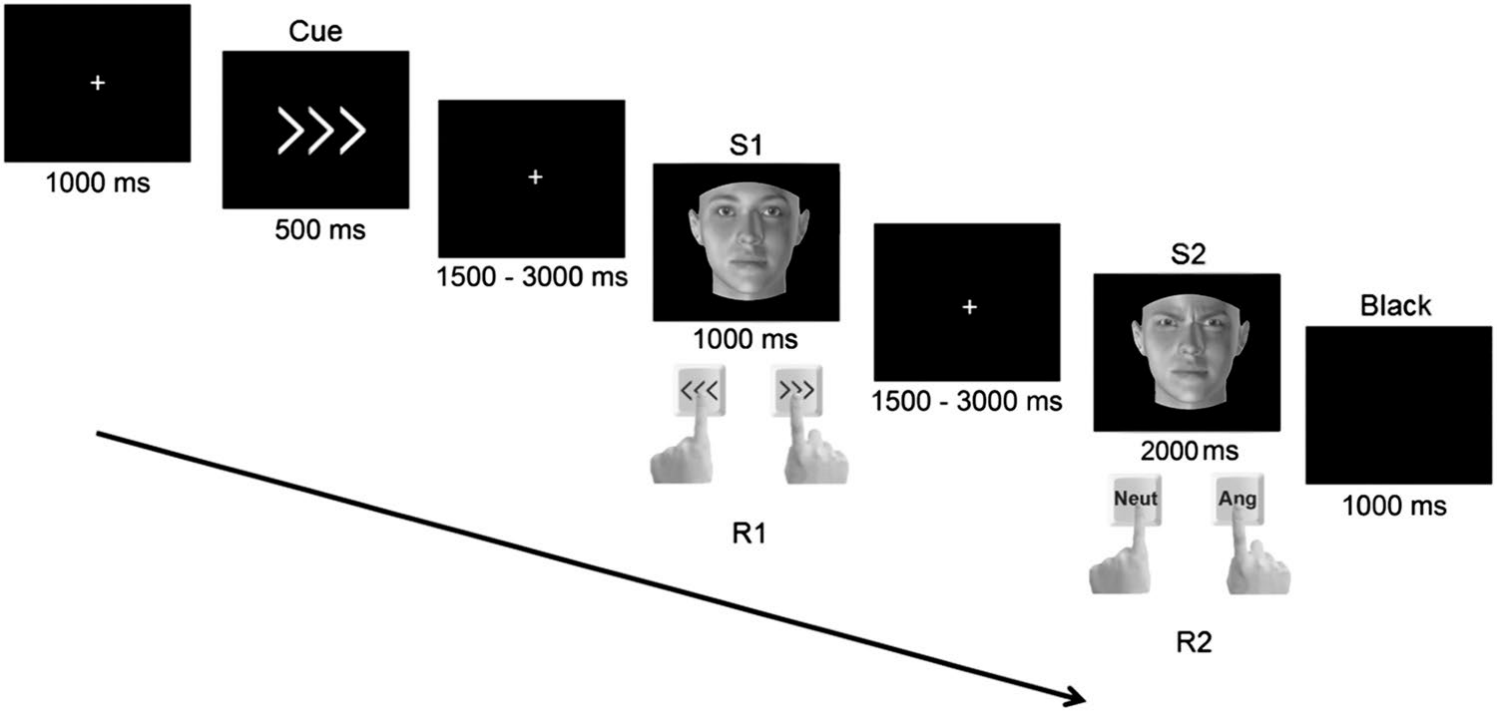
Overview of the displays and timing of events in the experiment. A cue (a left or right-pointing arrow) is presented announcing the response (R1) to be given to a first angry or neutral face (S1). Then a second angry or neutral face (S2) appears and participants have to answer (R2) given the emotion expressed by it.

Participants were comfortably installed in the scanner and were invited to perform the following task (see Fig. 6): A fixation cross appeared in the middle of the screen for 1000 ms. It was then replaced by a cue, represented by rows of 3 left- or right-pointing arrows, for 500 ms. Participants were instructed to look at the cue. Next, a second fixation cross was presented with a jitter (1500–3000 ms) and then S1 appeared on the screen. It could be a man or a woman with his/her face expressing anger or being neutral. Participants responded to that stimulus by giving the direction of the cue with a left or right button press using their respective index fingers. This response was thus independent of the face’s identity and emotion portrayed. After another 1500 to 3000 ms interval, S2 was presented for 2000 ms. This second face could vary from S1 depending on the facial emotion expressed. Participants responded to that stimulus according to the emotion of the face. Half of the participants responded with a right button press for an angry face and a left button press for a neutral face. The other half had opposite instructions. Finally, a blank screen appeared for 1000 ms before the next trial.

Feedback was added to the paradigm to ensure that participants answered correctly. First, participants were told to answer according to the cue when they did not respond or made a mistake in S1. When this situation happened, the trial started again from the beginning. Second, participants were asked to answer according to the emotion when they did not respond or made a mistake in S2. In this case, if they did not respond, participants started the trial again from the beginning, but if they made a mistake, they continued the trial.

The binding task comprised 128 trials composed of a factorial combination of the cue (left vs. right), emotion (angry vs. neutral) and identity of the face (two women vs. two men), as well as repetition versus alternation of the emotion. Our variables of interest were the emotion (S1 and S2 angry: sameemo vs. S1 and S2 neutral: sameneut vs. S1 angry and S2 neutral: diffemoneut vs. S1 neutral and S2 angry: diffneutemo) and the motor response which could be the same vs. different to S1 motor response. Each experimental condition was repeated 16 times to ensure enough trials for the analysis. Trials were presented in a pseudo-randomized order, in which the same experimental condition was never shown 3 times in a row. The task was divided into 3 blocks of approximately 43 trials (43, 43, and 42 for bloc 1, 2, and 3, respectively) and lasted about 30 min.

#### Finger localizer task

In order to locate the motor areas activated when responding with the right and left index fingers, participants performed the following task after the binding task: A fixation cross was displayed in the middle of the screen for 500 ms, replaced next for 1000 ms by the right cue used in the binding task. This sequence was repeated 20 times and participants had to press the right button when the cue appeared on the screen. After a 10 s break, 20 sequences in which participants had to respond with the left button followed. The whole sequence (right response – break – left response) was repeated 72 times for a total approximate duration of 5 min.

### Image acquisition

Data of the binding task and finger localizer were acquired at 3 T using a Siemens Trio System (Siemens, Erlangen, Germany) using a standard T_2_^*^-weighted gradient echo planar imaging sequence (3 mm^3^ voxel resolution, distance factor = 30%, field of view = 192 mm) in which a continuous whole-head acquisition of 36 slices aligned to the anterior and posterior commissure plane with time repetition [TR]/ time echo [TE] = 0.65/0.03 s. Finally, to obtain structural brain images from each participant, a high-resolution magnetization prepared rapid acquisition gradient echo T1-weighted sequence (TR = 1.9 s, TE = 2.32 ms, time to inversion [TI] = 900 ms) in sagittal orientation was performed.

### Behavioral analysis

To test our behavioral assumptions, that is, the binding between the emotion and motor response, and the different impact of emotional and neutral stimuli in the binding, we used the generalized linear mixed models (GLMMs) statistical method. GLMMs are interesting because they incorporate random effects and can be adapted to non-normal distributed data^61,62^. Moreover, with GLMMs the total variance of our data can be modeled without averaging the trials of individuals, as often done with classical analysis of variance.

Our fixed effects were emotion repetition (sameemo vs. sameneut vs. diffemoneut vs. diffneutemo) and motor response repetition (same vs. different). Our random effects were the participant and experimental block. We kept the latter as a random effect because there was a significant effect of adding it to our model, χ^2^(1, *N* = 19) = 27.75, *p* < 0.001. However, the gender, handedness, identity of the face in S2, as well as the response group (right index-anger group vs. right index-neutral group) random effect did not show a significant effect, and were thus removed from the model.

Trials with incorrect (6% of total trials), missing (>2 standard deviations above the mean; 4% of total trials), or anticipatory (<2 standard deviations under the mean; 0% of total trials) responses were excluded for the behavioral and neuroimaging analyses (described below).

### Image analysis

Neuroimaging data were pre-processed and analyzed using the Statistical Parametric Mapping software (SPM8; Wellcome Trust Centre for Neuroimaging, http://www.fil.ion.ucl.ac.uk/spm). In addition, functional connectivity was assessed using the Functional Connectivity (CONN) toolbox (http://web.mit.edu/swg/software.htm). CONN allows ROI-based analysis by grouping voxels into ROIs based upon Brodmann’s areas. Bi-variate correlations were calculated between each pair of ROIs as reflections of connections. All Brodmann areas were imported as possible connections for ROI sources chosen based on the results of our t-contrasts in the binding task and finger localizer. In order to validate the multiple comparisons, results were corrected using the false discovery rate (FDR) method with a significance level of 0.05 at the seed-level. Data pre-processing included realignment, coregistration to the anatomical image, segmentation, normalization to the Montreal Neurological Institute space using an EPI template (resampling voxel size: 2 × 2 × 2 mm), and spatial smoothing (nonisotropic Gaussian kernel of full-width at half-maximum 6 × 6 × 6 mm).

We used a general linear model for first-level statistical analyses, including boxcar functions defined by the onset and duration of S2’s faces, for the binding task, and the motor responses, for the finger localizer. These boxcar functions were convolved with a canonical hemodynamic response function. Separate regressors were created for each experimental condition, 8 in total: 2 motor response repetition conditions (same vs. different) x 4 emotion repetition conditions (sameemo vs. sameneut vs. diffemoneut vs. diffneutemo). Six motion correction parameters were included as regressors to minimize false-positive activations due to motion artifacts. Linear contrasts for the experimental conditions for each participant were taken to a second-level group analysis of variance, including the response group factor for the binding task.

#### Finger localizer analysis

T-contrasts comparing the right and left motor responses (*right* > *left* and *left* > *right*) were performed to locate the motor areas associated to the index finger responses and use them as a region of interests in the binding task analyses. All contrasts were thresholded at *p* < 0.001 (uncorrected) with a cluster extent of *k* = 38, corresponding, according to the AFNI 3dClustSim software (a non-parametric computation with 1000 iterations; software available at https://afni.nimh.nih.gov/afni/download), to a Family-Wise Error (FWE) correction for multiple comparisons of *p* < 0.05 at the cluster level.

#### Binding task analysis

Binding task analyses were conducted in 4 steps. Firstly, we were interested in the emotion influence in general, by comparing the activity due to an angry face in S2 to the activity associated to a neutral face in S2 using a t-contrast (*emotion* > *neutral*). Secondly, we wanted to investigate influence of the emotion-action binding. In order to do so, we compared situations of a complete repetition and alternation of the emotion and motor response to situations of a partial repetition of them (*complete repetition* + *alternation* > *partial repetition*; *complete repetition* > *partial repetition*; *complete alternation* >*partial repetition*). Thirdly, following the idea of a different impact of emotional and neutral information in the binding, we wanted to observe the brain areas more activated for a repetition of emotional than neutral faces when the motor response was also repeated (*same response* + *sameemotion* > *same response* + *sameneut*). All contrasts were thresholded at *p* < 0.001 (uncorrected) with a cluster extent of *k* = 23, corresponding to a Family-Wise Error (FWE) correction for multiple comparisons of *p* < 0.05 at the cluster level as calculated using AFNI 3dClustSim software. Finally, functional connectivity for our t-contrasts of interest (see above), as well as F-contrasts for a complete repetition, complete alternation and partial repetition of the emotion and motor response in the binding task was assessed on 11 selected ROI sources chosen based on their activation in the t-contrasts in the binding task (see Table 2) and the t-contrasts in the finger localizer (the right frontal precentral gyrus for the left motor responses and the left parietal postcentral gyrus for the right motor responses).

### Data availability

Under the Swiss guidelines of data protection (Ordinance HFV Art. 5), the datasets generated and analyzed during the current study can be made available from the corresponding author on a case by case basis.

## Electronic supplementary material

Supplementary materials
