# Supplementary material for: The behavioral and neural binding phenomena during visuomotor integration of angry facial expressions
Source: Sci Rep. 2018 May 2;8:6887. doi: 10.1038/s41598-018-25155-8 (PMC5931994; doi:10.1038/s41598-018-25155-8)
Supplement: Supplementary file 1 — Supplementary materials [file 41598_2018_25155_MOESM1_ESM.docx]

**Supplementary material**

The Behavioral and Neural Binding Phenomena during Visuomotor Integration of Angry Facial Expressions

Sélim Yahia Coll^1^, Leonardo Ceravolo^1^, Sascha Frühholz^2^ and Didier Grandjean^1^

^1^University of Geneva, Switzerland

^2^University of Zurich, Switzerland

Appendix A

Main Effects in the Reaction Time Analysis

Emotion repetition showed a significant main effect (*F*(3, 2152) = 17.55, *p* < 0.001, *R^2^_m_* = 0.02, *R^2^_c_* = 0.31). Participants were slower in the diffemoneut than in the diffneutemo (χ^2^(1, *N* = 19) = 33.11, *p* < 0.001) and the sameemo (χ^2^(1, *N* = 19) = 31.06, *p* < 0.001) conditions. Moreover, participants were slower in the sameneut than in the diffneutemo (χ^2^(1, *N* = 19) = 20.74, *p* < 0.001) and the sameemo (χ^2^(1, *N* = 19) = 18.87, *p* < 0.001) conditions. No significant difference was observed between the diffneutemo and the sameneut conditions (χ^2^(1, *N* = 19) = 1.30, *p* = 0.25), or between the diffneutemo and the sameemo conditions (χ^2^(1, *N* = 19) = 0.02, *p* = 0.89).

The motor response repetition main effect was not significant (*F*(1, 2152) = 0.78, *p* = 0.38, *R^2^_m_* < 0.001, *R^2^_c_* = 0.30).

Appendix B

Accuracy Analysis

Neither a significant main effect of the emotion (χ^2^(3, *N* = 19) = 5.67, *p* = 0.13, *R^2^_m_* < 0.01, *R^2^_c_* = 0.09) nor motor response (χ^2^(1, *N* = 19) = 0.01, *p* = 0.91, *R^2^_m_* < 0.001, *R^2^_c_* = 0.08) repetition was obtained. However, a significant interaction between the emotion and motor response repetition was observed (χ^2^(1, *N* = 19) = 32.95, *p* < 0.01, *R^2^_m_* = 0.08, *R^2^_c_* = 0.16). Participants were significantly more accurate in the diffemoneut (z=2.93, *p* < 0.01) and diffneutemo (z=2.69, *p* < 0.01) condition for a motor response alternation. Moreover, they were significantly more accurate in the sameneut condition (z=-3.25, *p* < 0.01) for a motor response repetition, but only marginally in the sameemo condition (z=-1.76, *p* = 0.08).

Appendix C

Binding task activations regarding the finger localizer


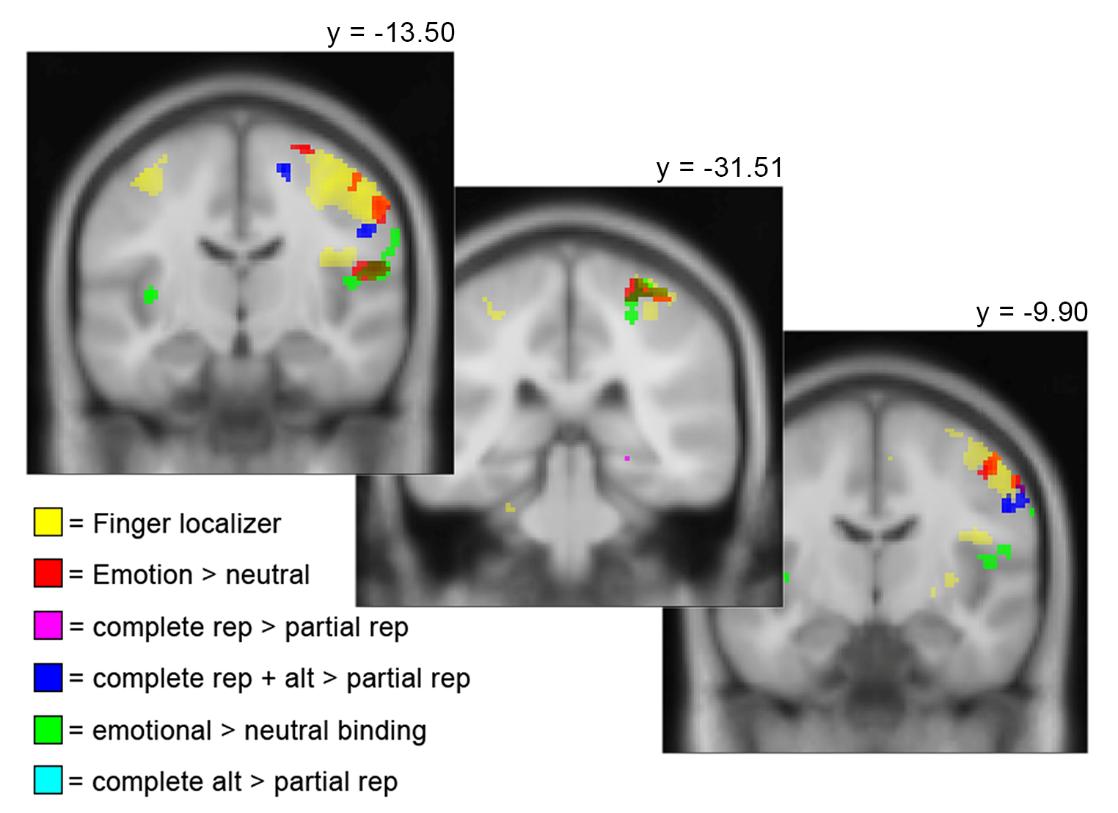


In yellow, *right > left finger* and *left > right finger* t contrasts. In red, *emotion > neutral* contrast. In pink, *complete repetition > partial repetition* t contrast. In dark blue, *complete repetition + alternation > partial repetition* t contrast. In green, *same response + sameemotion > same response + sameneut* t-contrast. In light blue, *complete alternation > partial repetition* t contrast.
